# Exercise alone or combined with functional foods in aging and age-related diseases: targeting DNA damage, repair pathways, and genome integrity

**DOI:** 10.3389/fnagi.2026.1772217

**Published:** 2026-08-14

**Authors:** Ying Ma, Xuejun Li, He Chen, Rongchang Fan

**Affiliations:** 1 School of Sports and Music, Central South University of Forestry and Technology, Changsha, Hunan, China; 2 Department of Physical Education, Central South University, Changsha, Hunan, China; 3 College of Physical Education, Sangmyung University, Seoul, Republic of Korea; 4 Ministry of Sports, Nanjing Railway Vocational and Technical College, Nanjing, Jiangsu, China

**Keywords:** aging, CNS-related disorders, DNA repair, exercise, functional foods

## Abstract

Aging is fundamentally associated with the gradual accumulation of DNA damage, which is instrumental in the initiation and advancement of age-associated pathologies, including neurodegenerative conditions such as Alzheimer’s disease and Parkinson’s disease. Recent studies indicate that lifestyle modifications—especially the consumption of functional foods and participation in consistent physical exercise—can influence cellular and molecular processes related to DNA repair, reduction of oxidative stress, and maintenance of genomic integrity. This review consolidates contemporary research regarding how particular bioactive compounds (e.g., polyphenols, omega-3 fatty acids, and flavonoids) present in functional foods, in conjunction with diverse exercise interventions, affect DNA repair processes and diminish genotoxic stress. We examine evidence from exercise-only, nutrition-only, and combined lifestyle interventions, with particular attention to whether combined interventions show additive, complementary, or genuinely synergistic effects on oxidative stress, inflammatory responses, epigenetic aging, and DNA repair-related pathways. Particular emphasis is placed on the susceptibility of the brain to DNA damage and the significance of these interventions in safeguarding neuronal integrity and cognitive capabilities. By synthesizing evidence from molecular, animal, and human research, this review underscores the therapeutic promise of functional foods and physical activity as readily available, non-pharmacological approaches to ameliorate genomic integrity and postpone the onset or progression of aging-related and neurodegenerative disorders. In conclusion, we address prevailing limitations, deficiencies in translational research, and prospective avenues for personalized lifestyle medicine aimed at enhancing DNA integrity.

## Introduction

The basic units of the nervous system are neurons that have finished dividing, responsible for regulating essential bodily functions like movement, respiration, and heart function, as well as more complex cognitive abilities like memory and attention (Khan et al., 2020). However, since neurons are predominantly a finite and restricted asset, they are required to carry out these important functions while simultaneously preserving their structural and genetic well-being throughout their extended lifespan. To endure the unstoppable progression of time, neurons possess reliable and effective mechanisms to respond to damage in their DNA, (DDR) pathways. If these pathways are faulty, it can lead to harmful changes in the genetic code, disruptions in gene expression, and a buildup of unaddressed DNA damage (Hoeijmakers, 2009; Madabhushi et al., 2014; Chow and Herrup, 2015; Tubbs and Nussenzweig, 2017; Welch and Tsai, 2022; Shou et al., 2024). In conclusion, these offending terms ultimately drive the determination of cellular destiny toward programmed cell death, cellular aging, or unregulated cell proliferation, which are all key indicators of age-related disorders (Hoeijmakers, 2009; Madabhushi et al., 2014; Chow and Herrup, 2015; Tubbs and Nussenzweig, 2017; Welch and Tsai, 2022). The significance of neuron viability can be fully understood when we consider the destructive impact of neurodegenerative disorders, which result in the loss of memories, motor skills, and independence. In 2017, these circumstances are presently identified as the third most prominent cause of death in the United States and the fifth globally (Feigin et al., 2021).

Rigorous research has definitively proven a significant connection between the deterioration of DNA and the appearance of neurodegenerative illnesses. Numerous studies have consistently confirmed that DNA damage is often an initial sign of neurological disorders, indicating that it may be the underlying cause of harmful influences causing the onset of these ailments (Chow and Herrup, 2015; Simpson et al., 2015; Simpson et al., 2016; Shanbhag et al., 2019). In recent times, a plethora of discoveries have provided a better understanding of how DNA damage can result in impairment in neurons. These insights have been derived from studies of both genetic disorders related to DNA damage repair and experimental models of neurodegenerative diseases associated with aging. Furthermore, with the development of sequencing methods for identifying DNA damage, alterations, and changes, we are only just starting to understand the importance of where a lesion is located within the genome and how it impacts neuronal functioning and, ultimately, degeneration (Lodato et al., 2015; Lodato et al., 2018; Wei et al., 2016; McConnell et al., 2017; Reid et al., 2021; Rodin et al., 2021; Wu et al., 2021). Ultimately, although neuroinflammation and DNA damage are widely accepted as key elements and catalysts in the process of neurodegeneration, a thorough understanding of the interplay between the two remains to be achieved. To fully comprehend this dynamic, principles from the field of senescence cell biology are aiding us in deciphering how these factors may influence and impact each other (Zhang et al., 2019; Gillispie et al., 2021; Chow et al., 2019; Musi et al., 2018). In this section, we will examine the latest progress made in various fields of disease research and its impact on enhancing our comprehension of the functioning and degradation of neurons.

There is recognition that outside influences can potentially protect or delay harm done to DNA, such as participating in physical exercise (Soares et al., 2015). Multiple studies have confirmed that participating in physical exercise brings positive effects on brain protection and improves overall performance. This is accomplished by promoting plasticity, learning, memory, and cognition, while also regulating the cellular redox state (van Praag et al., 2005; Tuon et al., 2015; Baek, 2016; Vilela et al., 2017; Pinho et al., 2019). This rule prompts an increase in the body’s ability to produce antioxidants, ultimately leading to a decrease in the levels of harmful reactive oxygen molecules and the resulting harm to a range of biological molecules including DNA, lipids, and proteins (Radak et al., 2016; Radak et al., 2013). Various forms of physical exercise can alter the balance of redox reactions in the brain in distinct manners. Furthermore, the impact of both temporary and consistent physical activity on the state of redox reactions are different (Shahandeh et al., 2013). For example, when faced with an unexpected increase in physical exertion, the body struggles to adapt. Nevertheless, regular physical activity initiates changes in redox signaling, which impacts the functioning of many different bodily processes (Radak et al., 2016). The present review article is warranted due to the fact that, although numerous investigations have examined the distinct influences of functional foods and physical exercise on the aging process and associated diseases, there exists a significant deficiency in exhaustive reviews that amalgamate these two pivotal lifestyle determinants specifically as modulators of DNA damage in aging-related pathologies. Currently available reviews predominantly concentrate on either dietary interventions or physical activity in isolation, failing to adequately address their collective effect on genomic stability and the fundamental molecular mechanisms involved. This article addresses this deficiency by integrating emerging evidence regarding the independent, additive, and potentially complementary roles of functional foods and exercise as modulators of genomic stability. The objective of the present investigation is to meticulously examine and integrate existing empirical data regarding the contributions of functional foods and physical activity in the modulation of DNA damage linked to aging-associated pathologies, with specific attention to whether combined interventions produce additive or complementary effects beyond either intervention alone and the fundamental molecular processes involved, with the intention of guiding forthcoming preventive and therapeutic interventions aimed at promoting aging.

## Literature search strategy and review approach

This article was designed as a structured narrative review rather than a systematic review or meta-analysis. A targeted literature search was performed in PubMed/MEDLINE, Scopus, Web of Science, and Google Scholar to identify peer-reviewed studies addressing DNA damage, DNA repair, aging, neurodegenerative disease, exercise, functional foods, and nutritional supplementation. The search combined terms related to genome integrity (“DNA damage,” “DNA repair,” “base excision repair,” “nucleotide excision repair,” “non-homologous end joining,” “homologous recombination,” “PARP1,” “8-OHdG,” “micronuclei,” “telomere,” and “genomic stability”) with terms related to lifestyle interventions (“exercise,” “aerobic training,” “resistance training,” “high-intensity interval training,” “physical activity,” “functional foods,” “polyphenols,” “flavonoids,” “omega-3,” “vitamin D,” “vitamin E,” “creatine,” and “L-arginine”) and disease contexts (“aging,” “Alzheimer’s disease,” “Parkinson’s disease,” “Huntington’s disease,” “amyotrophic lateral sclerosis,” “frontotemporal dementia,” and “neurodegeneration”).

Priority was given to original mechanistic studies, controlled human intervention studies, animal studies with direct DNA damage or repair endpoints, and recent high-quality reviews relevant to aging and neurodegenerative disorders. Studies were excluded when they were not directly related to DNA damage, DNA repair, genome integrity, aging biology, exercise physiology, functional foods, or neurodegenerative disease mechanisms. Because this was not a systematic review, formal risk-of-bias scoring and meta-analysis were not performed. Instead, the evidence was narratively synthesized, and mechanistic claims were interpreted according to whether they were supported by human intervention data, animal models, cellular studies, or indirect mechanistic evidence.

## DNA damage response and repair mechanisms

DNA can be damaged through natural cellular processes (endogenous damage) or outside influences (exogenous damage). Both of these types of damage can be caused by various agents or mechanisms. Endogenous damage involves changes to the DNA such as mismatched bases, loss of nitrogenous bases due to hydrolysis, alkylation (such as methylation), oxidation, and breaks in the DNA. Exogenous damage, on the other hand, includes processes like dimerization, the formation of large adducts, external alkylation, the presence of free radicals, damage caused by external factors, heat damage, and changes in the structure of nitrogenous bases (such as keto-enol and amino-imino isomerism) (Wang, 2001; Chakarov et al., 2014). Practices that shape one’s daily routine, such as engaging in physical activity, provide protection for the nervous system and enhance brain functioning through memory, learning, and cognitive improvements, while also balancing the body’s cellular oxidation. Various mechanisms contribute to the positive effects of exercise on the brain, including a decrease in the production of harmful oxidants, an increase in antioxidant capacity, and a subsequent reduction in damage to nuclear DNA. Furthermore, physical exercise has the potential to aid in the repair of DNA damage. Lifestyle choices, specifically smoking, body weight, and level of physical activity, have a significant impact on the levels of blood serum markers for telomere dysfunction and DNA damage, regardless of age, and these markers are correlated with indicators of cellular aging in T-lymphocytes found in the blood (Song et al., 2010). We still do not know the specific neuroplasticity molecules that are activated by various types of physical exercise (Vilela et al., 2020). To this day, it has been proven that physical activity creates an ideal setting for neuroplasticity in the primary motor cortex and other brain areas crucial for motor control. This process, induced by exercise, promotes the development and function of motor skills. The evidence supporting the relationship between exercise and neuroplasticity is further reinforced by studies showing an increase in neuroplasticity after non-invasive brain stimulation following exercise. The ability of exercise-induced plasticity to aid in motor learning is particularly beneficial for maintaining motor function in older individuals and for improving motor skill re-acquisition in stroke or Parkinson’s patients who struggle with acquiring and retaining motor abilities. However, there are still gaps in our understanding that require further investigation (Nicolini et al., 2021).

**Figure 1:**
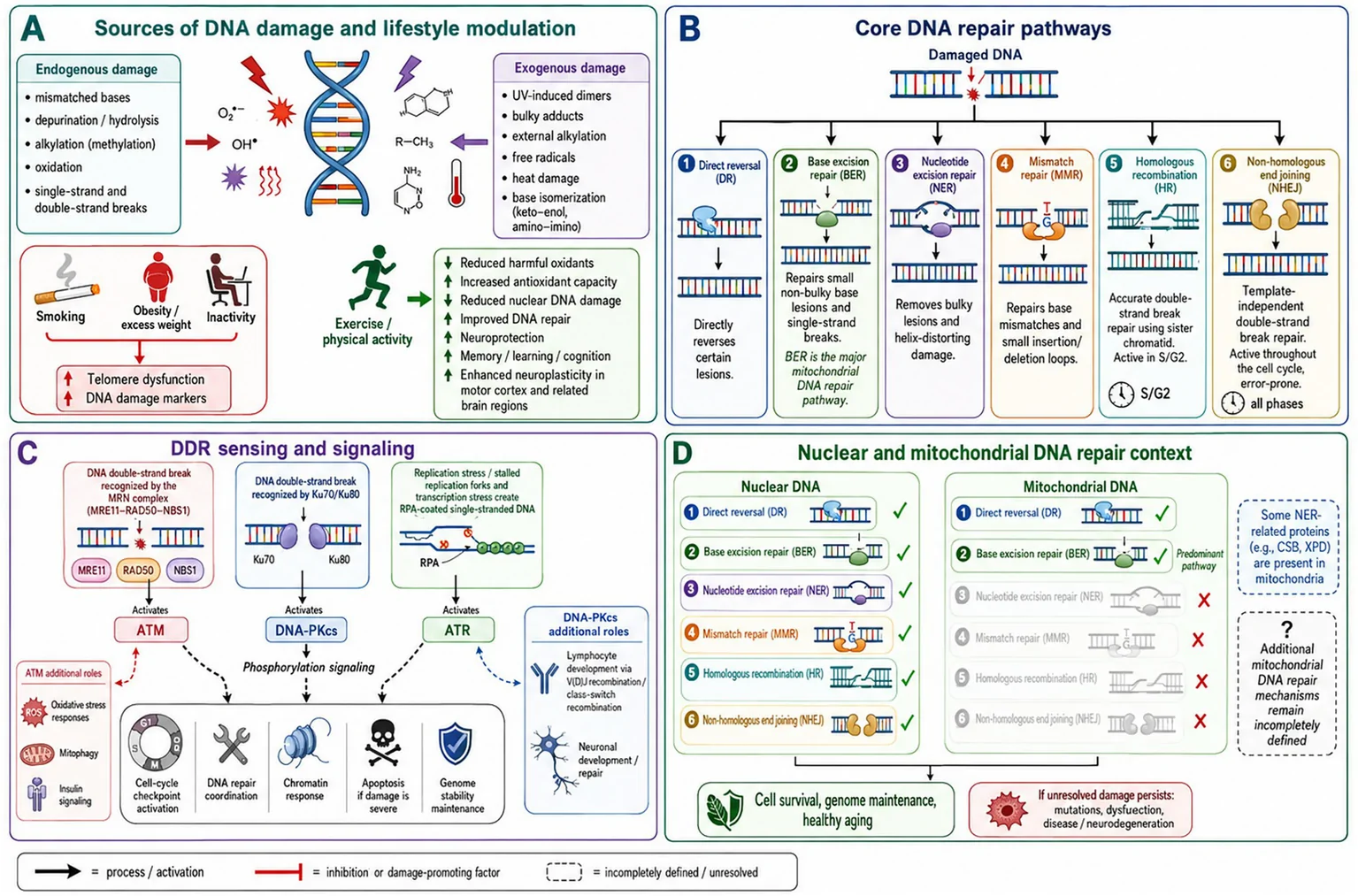
Overview of DNA damage, DNA repair pathways, and DNA damage response (DDR) signaling. **(A)** Sources of DNA damage and lifestyle modulation; **(B)** core DNA repair pathways; **(C)** DDR sensing and signaling; and **(D)** nuclear and mitochondrial DNA repair context. Endogenous and exogenous insults generate diverse DNA lesions that are processed through direct reversal, base excision repair (BER), nucleotide excision repair (NER), mismatch repair (MMR), homologous recombination (HR), and non-homologous end joining (NHEJ). Ataxia-telangiectasia mutated (ATM), ataxia-telangiectasia and Rad3-related (ATR), and DNA-dependent protein kinase catalytic subunit (DNA-PKcs) coordinate checkpoint signaling, chromatin responses, DNA repair, apoptosis, and genome-stability maintenance. The figure also contrasts nuclear and mitochondrial DNA repair contexts, emphasizing BER as the predominant mitochondrial repair pathway and highlighting unresolved questions regarding additional mitochondrial DNA repair mechanisms.

The DDR mechanism discovered within mammalian cells is an intricate and sophisticated process that efficiently manages the cell’s response to any harm inflicted on its DNA (Zhou and Elledge, 2000). The DDR (Figure 1) relies on six primary DNA repair mechanisms, which are essential for its functioning. These include mismatch repair (MMR), direct reversal (DR), nucleotide excision, and base excision repair (BER) repair (NER), which all work to eliminate damaged or mismatched nucleotides. There are also two pathways for repairing double-strand breaks (DSBR): non-homologous end joining (NHEJ) and homologous recombination (HR) (Yu et al., 1999). NER is an extremely adaptable process that can effectively address a range of DNA damage, including the formation of major defects in DNA caused by exposure to hazardous environmental substances and mistakes during cell division (Nouspikel, 2009). The main function of BER is to rectify faulty DNA bases that do not result in palpable alterations in the structure of DNA (Nilsen and Krokan, 2001). The BER proteins are involved in fixing single-strand breaks (SSBs), while MMR is responsible for addressing minor insertion/deletion loops and incorrect nucleotide pairings (Hoeijmakers, 2001). BER is widely recognized as the leading strategy for mitigating damage to mitochondrial DNA (Fontana and Gahlon, 2020). The traditional MMR, DSBR, and NER pathways are not functional within the mitochondria due to the absence of numerous critical enzymes. Nevertheless, certain proteins such as CSB and XPD from the NER pathway are present within the mitochondria (Liu et al., 2015; Aamann et al., 2010). It remains necessary to demonstrate whether there exist mitochondrial DNA repair mechanisms beyond BER, or if alternative strategies are utilized to ensure appropriate maintenance of mitochondrial DNA (Zhao and Sumberaz, 2020). HR is a process that mends breaks in both strands of DNA and also repairs DNA crosslinks between strands. Since HR relies on a matching sister chromatid as a guide for mending, it is only effective during the S and G2 stages of the cell cycle and is recognized as a trustworthy method for repairing DNA. On the other hand, NHEJ does not depend on homology and can immediately patch up breaks that have blunt ends. In every phase of the cell cycle, NHEJ remains continuously active (Jackson and Bartek, 2009; Mao et al., 2008). Since NHEJ does not make use of a template during the repair process and may involve removing portions of the ends to ready them for relegation, there is a possibility of introducing mutations. This is why NHEJ is commonly known as “error-prone.” (Brandsma and Gent, 2012; Her and Bunting, 2018). In a manner akin to nuclear DNA, mitochondrial DNA is also susceptible to influences from both internal and external sources. Along with mechanisms for repairing damaged DNA, the process known as DDR includes the triggering of signaling pathways that work together to guide the cellular reactions to DNA harm (see Lukas et al., 2011) or (Nair et al., 2017).

DNA-PK, ATM, and ATR have a critical part in regulating the subsequent DDR signaling pathways by activating phosphorylation on a large number of enzymes (Blackford and Jackson, 2017). The catalytic subunits of both ATM and DNA-PK also referred to as DNA-PKcs, are triggered into action in the presence of DNA DSBs. However, their activation is initiated by separate sensor complexes responsible for detecting DNA damage. After being triggered by the MRN complex, which is made up of RAD50, NBS1, and MRE11, ATM carries out a process called phosphorylation on a variety of other proteins that play a role in DNA DDR, as previously stated (Blackford and Jackson, 2017; Lavin, 2008; Shiloh, 2003; Ciccia and Elledge, 2010; Banin et al., 1998; Bassing et al., 2003). Despite its main purpose, ATM also has a critical function in the activation of multiple proteins that are involved in various functions within the cell. Alongside its involvement in DNA repair, ATM also serves as an important controller for mitophagy, responses to oxidative stress, and signaling for insulin (Ditch and Paull, 2012; Pizzamiglio et al., 2020). The process of non-homologous end joining is reliant on the activation of DNA-PKcs, which occurs when Ku80 and Ku70 molecules bind to DNA to form a complex. This component is crucial for the functioning of DNA-PKcs (Blackford and Jackson, 2017; Anisenko et al., 2020). By taking part in NHEJ, DNA-PKcs contribute to the development of lymphocytes and neurons, as well as serve a purpose in repairing DNA after the integration of HIV-1 (Anisenko et al., 2020; Alt et al., 2013; Woodbine et al., 2013). As lymphocytes undergo development, they intentionally create DSBs in specific areas of the T cell receptor genes and immunoglobulin. The activity referred to as V(D)J recombination and class-switch recombination is crucial in producing a wide range of immune receptors. The act of repairing the DSBs is imperative in accurately connecting the V(D)J recombination intermediates, which is aided by the DNA-PK enzyme through a process called NHEJ (Alt et al., 2013).

The process of repairing damaged DNA, specifically DSBs, which can happen during the formation of nerve cells, involves the use of DNA-PK and other DNA repair components. Mutations in the DNA-PKcs gene can directly affect the efficiency of non-homologous end joining (NHEJ) repair, which can have serious consequences on neurological function (Woodbine et al., 2013; Jiang et al., 2015). ATR is closely related to DNA-PKcs and ATM (Blackford and Jackson, 2017). ATR involve in the activation of single-stranded DNA segments that are shielded by Replication Protein A (RPA). This happens as a reaction to various forms of genetic stress, such as a halted duplication process and transcription. In addition to its primary role of triggering the DNA damage checkpoint, ATR also aids in promoting continuous DNA replication (Brown and Baltimore, 2003).

## DNA damage in CNS-related disorders

After noticing an increase in oxidative DNA damage in both nuclear and mitochondrial DNA samples extracted from various brain regions of patients with neurodegenerative diseases, a considerable amount of research has been focused on identifying markers of DNA damage in neuronal samples and tissues from patients at various stages of neurodegeneration. These studies have provided evidence that oxidative DNA damage is among the first signs of neurodegeneration, and have also uncovered cytogenetic damage, such as a tendency for chromosome 21 malsegregation in Alzheimer’s disease (AD). While the cause or consequence of this DNA damage is still under debate, it is likely that both are true. Nevertheless, it is widely accepted that oxidative DNA damage, particularly in the mitochondrial DNA, is an early event in the progression of neurodegeneration, and can contribute to the disruption of mitochondrial function and perpetuation of oxidative stress in neurons. Ultimately, this can lead to the development of neurodegenerative diseases (Coppedè and Migliore, 2015).

Parkinson’s disease (PD) is the second-most common degenerative illness impacting the nervous system, following AD. It is estimated to impact over 3% of individuals aged 80 and above (Poewe et al., 2017; Savica et al., 2013; Twelves et al., 2003). Individuals suffering from this condition exhibit a decline in nerve cells located in the substantia nigra, as well as the presence of intracellular Lewy bodies predominantly composed of *α*-synuclein accumulations (Poewe et al., 2017). The primary indicators of PD’s are uncontrollable shaking, sluggish motor functions, and tense muscles. These manifestations typically manifest themselves in individuals over the age of 50 (Van Den Eeden et al., 2003). Malfunctions in DNA repair lead to the early development of key aspects of PD, including problems with mitochondria, disruptions in the dopaminergic system, and impaired regulation of protein quality (Poewe et al., 2017). The key connection between reduced DNA repair and PD arises from *α*-synuclein, which has been pinpointed as the trigger for activating the critical DNA damage repair signaling enzyme called ataxia telangiectasia mutated (ATM), as well as two associated elements, namely p53-Binding Protein 1 (53BP1) and phospho-histone H2AX (γH2AX) (Milanese et al., 2018). The introduction of antioxidants played a crucial role in restoring this activation, indicating that oxidative stress was only partially responsible. It has been recently noted that PARP1 participates in the process of PD (Kam et al., 2018). The excessive production of PAR by PARP1 is accountable for the deterioration of dopaminergic neurons. Higher levels of poly (ADP-ribose) lead to a faster buildup of *α*-synuclein, which causes the creation of a denser and altered protein that is significantly more harmful, exhibiting a potency 25 times stronger. This amplified toxicity can be mitigated by hindering and reducing PARP1 activity. Both PAR and α-synuclein have been detected in CSF and other biological fluids, making them potential indicators of PD development (Kam et al., 2018; Kruse and Mollenhauer, 2019; Atik et al., 2016).

NER is extremely important for preserving the overall health and functioning of the dopaminergic system (Sepe et al., 2016). Fibroblasts of patients with PD lack the ability to effectively carry out NER, while mice carrying mutations in the Ercc1 gene display deficiencies in their dopaminergic neurons, suffer from impaired mitochondrial function, and experience heightened phosphorylation of *α*-synuclein. AD is a degenerative condition that gradually worsens over time and targets around 47 million people across the globe (Wimo and Prince, 2010). According to projections, the number is anticipated to rise to 74.7 million by 2030 (Wimo and Prince, 2010). The defining features of AD include difficulties with recalling and reasoning, the onset of dementia, and the decline of neuronal cells. Those with this disease typically have an accumulation of Aβ plaques outside the brain cells and tangled tau proteins inside the cells. Modifications in the amounts of Aβ-42 and phosphorylated tau found in the cerebrospinal fluid are clear indications of the existence of amyloid plaques and impairment to nerve cells (McKhann et al., 2011).

As individuals grow older, they become more susceptible to developing AD due to the accumulation of oxidative stress. Numerous studies suggest that this oxidative stress leads to reduced brain function and the degeneration of nerve cells, brought on by the oxidation of nucleic acids (Lovell and Markesbery, 2007; Hegde et al., 2012; Moreira et al., 2008). Extensive studies have demonstrated that AD results in a significant rise in oxidative stress that damages DNA. In order to counteract this damage, the body utilizes BER mechanism. The process, which has been extensively studied by Wallace and colleagues, focuses on repairing single-strand breaks in DNA caused by deamination, oxidation, depurination, or alkylation (Wallace, 2014). As the extent of harm surpasses the possibility of restoration, the aged brain experiences cellular senescence, apoptosis, or genome mutation, which are more likely to occur (Campisi, 2013; Smith et al., 2000; Huang et al., 2016).

**Figure 2:**
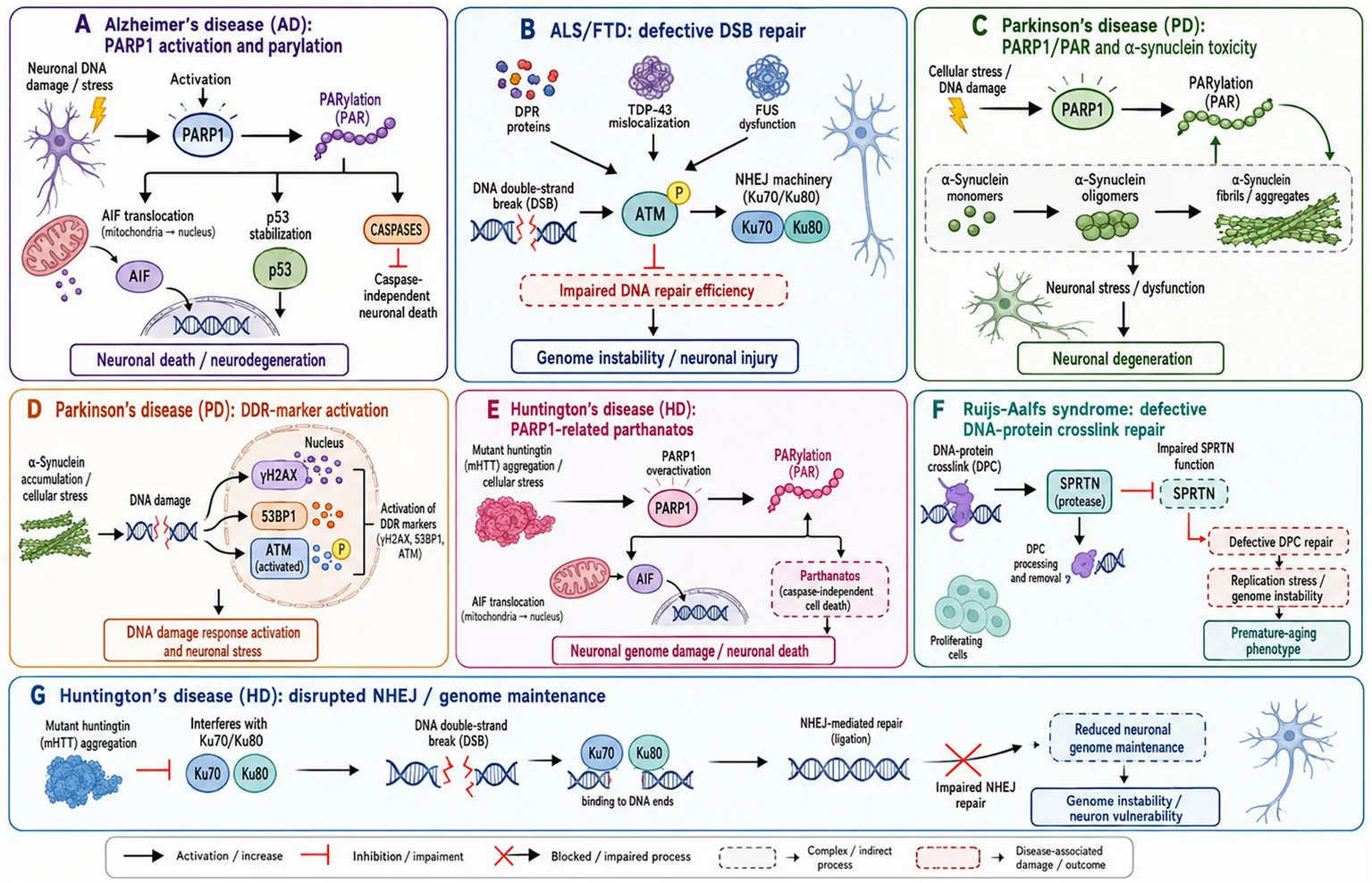
DNA repair defects and genome-instability mechanisms in neurodegenerative and premature-aging disorders. **(A)** AD-related PARP1 activation and PARylation can promote AIF translocation, p53 stabilization, and caspase-independent neuronal death. **(B)** ALS/FTD-related DPR proteins, TDP-43 mislocalization, and FUS dysfunction impair ATM/NHEJ-associated double-strand break repair. **(C,D)** In PD, PARP1/PAR signaling accelerates *α*-synuclein toxicity, while α-synuclein-associated stress activates DDR markers such as γH2AX, 53BP1, and ATM. **(E,G)** In Huntington’s disease, PARP1-related parthanatos and mutant huntingtin-mediated disruption of Ku70/Ku80 impair neuronal genome maintenance. **(F)** Ruijs-Aalfs syndrome illustrates defective SPRTN-dependent DNA-protein crosslink repair. Detailed mechanisms are discussed in the main text. AD, Alzheimer’s disease; ALS, amyotrophic lateral sclerosis; DDR, DNA damage response; DPR, dipeptide repeat; FTD, frontotemporal dementia; HD, Huntington’s disease; NHEJ, non-homologous end joining; PD, Parkinson’s disease.

The examination of BER gene levels in blood samples, brain tissue, and cerebrospinal fluid from healthy individuals (HC) and those with AD or mild cognitive impairment (MCI) was conducted. In individuals with mild cognitive impairment (MCI) and early-stage AD, the levels of mRNA for OGG1 were reduced, while the levels of mRNA for PARP1, a protein essential for DNA repair, were elevated in their blood. This was observed irrespective of the concentration of Aβ-42 and tau proteins in the cerebrospinal fluid. However, in healthy controls (HC), there was no similar change, indicating disrupted DNA repair in those who are susceptible to developing AD (Lillenes et al., 2016). This data was acquired using the non-invasive technique of venepuncture, indicating that OGG1 and PARP1 could potentially serve as useful biomarkers in clinical settings for detecting AD at an early stage (Lillenes et al., 2016). The possibility exists for nuclear DNA damage to increase the production of these DNA damage proteins. The accuracy and effectiveness of these indicators in other forms of dementia, aside from AD, will require further examination (Watabe-Rudolph et al., 2012). Studies have confirmed that elevated levels of PARP1 significantly enhance the activity of AMP protein kinase. This leads to the transfer of apoptosis inducing factor from the mitochondria to the nucleus, ultimately resulting in the death of the cell through a pathway that does not involve caspase (Narne et al., 2017). Furthermore, the process of PARylation has been demonstrated to enhance the stability of activated p53, resulting in a heightened level of cellular apoptosis brought about by the elevation of pro-apoptotic protein levels (Figure 2A) (Martire et al., 2015).

Amyotrophic lateral sclerosis (ALS) is a condition that impacts the nervous system and specifically the motor neurons, and has a prevalence of approximately 0.003% in Europe and the United States (Kiernan et al., 2011). ALS is characterized by the degeneration and dysfunction of both the lower motor neurons in the spinal cord and the upper motor neurons in the motor cortex region (Chio et al., 2009). FTD, the abbreviation for frontotemporal dementia, is the second most widespread type of dementia after AD. This progressive condition is marked by the deterioration of spindle neurons in the frontal and temporal areas of the brain (Couratier et al., 2017). Both ALS and FTD have a similar underlying cause. The combined ALS/FTD condition can be caused by genetic mutations in several genes including TARDBP, FUS, UBQLN2, VCP, SOD1, and SQSTM1/p62 (Brettschneider et al., 2012; Ji et al., 2017; Konopka and Atkin, 2018). TDP-43 controls the expression, modification, and movement of RNA, given its capacity to adhere to both RNA and DNA (Deshaies et al., 2018). Additionally, a recent investigation has supplied proof of the involvement of TDP-43 in the restoration of DNA double-strand breaks (DSBs). Specifically, researchers have noted the presence of TDP-43 at sites of DNA damage and its interaction with different proteins responsible for non-homologous end joining (NHEJ) repair, namely Ku70, LIG4, 53BP1, and XRCC4, in neurons (Figure 2B) (Mitra et al., 2019).

Under normal bodily conditions, TDP-43 is primarily located in the nucleus. The presence of nuclear localization and export signals enables it to effectively travel between the nucleus and cytoplasm (Tamaki et al., 2018). When TDP-43 is not in its proper location, it can be extremely damaging to neurons and has been connected to the process of ALS/FTD (Igaz et al., 2011; Barmada et al., 2010; Archbold et al., 2018). Altered TDP-43 results in a shift of its location to the cytoplasm, a reduction in its presence in the nucleus, and the clumping together of this protein into stress granules (Figure 2B) (Tamaki et al., 2018). Ninety-seven percent of individuals with ALS and approximately 50 % of individuals with FTD exhibit ubiquitinated inclusions containing misfolded TDP-43 in their cytoplasmic aggregates (Deshaies et al., 2018; Igaz et al., 2011; Archbold et al., 2018; Neumann et al., 2006). The finding of unusual accumulations of the TDP-43 protein has been deemed a critical element in the onset of numerous degenerative conditions affecting the nervous system (Gao et al., 2018). Significant rises in the levels of TDP-43 were noted in the blood of individuals with AD, FTD, and ALS, as compared to both healthy individuals and those without any form of dementia (Foulds et al., 2008; Verstraete et al., 2012; Junttila et al., 2016; Majumder et al., 2018). Scientists have found that the quantities of TDP-43 present in the blood plasma of people affected by AD and FTD are closely correlated to the amounts detected in their brain tissue after their passing. However, the amounts of TDP-43 in the CSF differ among individuals with ALS, with those with lower levels having a poorer prognosis (Noto et al., 2011; Feneberg et al., 2018).

While TDP-43 is present in every body tissue, the variants linked to ALS and FTD are solely found in the brain, presenting difficulties in identifying it in blood samples due to the barrier separating the brain from the blood (Igaz et al., 2011; Feneberg et al., 2018). On the other hand, CSF provides a direct pathway to the brain, enabling a more accurate assessment of TDP-43 levels specific to the brain. Despite its advantages, several limitations hinder the measurement of TDP-43 in the CSF, including lower concentrations and the decreased binding affinity of TDP-43 antibodies in the presence of elevated levels of albumin and immunoglobulins (Feneberg et al., 2018). More studies are required in order to fully ascertain the capabilities of TDP-43 as a biomarker, as its low level of diagnostic precision and considerable discrepancies in measurement must be taken into account (Feneberg et al., 2018). Individuals with ALS/FTD and genetic alterations in the FUS gene experience the accumulation of FUS protein clumps within stress granules in the cytoplasm of motor neurons, mirroring the pattern seen with TDP-43 protein pathology (Naumann et al., 2018). Recent discoveries indicate a connection between the grouping of FUS protein and impeded restoration of DNA wounds. It has been noted that the presence of FUS at damaged DNA locations is facilitated by the involvement of PARP1 (Naumann et al., 2018; Mastrocola et al., 2013). Modifications to the nuclear localization signal (NLS) of FUS lead to a higher level of damage to DNA found in the spinal cord of individuals suffering from ALS (Qiu et al., 2014). The results align with a recent investigation that revealed heightened levels of DSB indicators in FUS-ALS neural cells. This suggests that the rise in DNA damage precedes the formation of mutant FUS aggregates in the cytoplasm, hinting that deficiencies in DDR mechanisms could be a contributing factor to FUS-ALS characteristics. In addition, inhibiting PARP1 activity led to a reduction in the build-up of FUS protein at sites of DNA damage, causing FUS to be mislocalized in the cytoplasm and aggregate. However, targeting PARG, an enzyme that breaks down PAR chains, was able to counteract the detrimental effects of FUS-ALS. This was accomplished by promoting FUS binding to the damaged areas and reducing its accumulation in the cytoplasm (Naumann et al., 2018).

## Huntington’s disease and Ruijs-Aalfs syndrome

Although the preceding discussion focuses primarily on AD, PD, and ALS/FTD, additional DNA-repair defects are also relevant to Huntington’s disease and Ruijs-Aalfs syndrome. In Huntington’s disease, mutant huntingtin contributes to genomic instability by interfering with Ku70-mediated non-homologous end joining (NHEJ), thereby impairing DNA-PK-dependent double-strand break repair and promoting DSB accumulation in neurons (Enokido et al., 2010). This mechanism complements PARP1-linked neurotoxicity, in which excessive PARP1 activation can drive PAR accumulation, AMPK activation, mitochondrial AIF translocation, and caspase-independent neuronal death through parthanatos-related pathways (Narne et al., 2017; Martire et al., 2015). The convergence of impaired NHEJ, PARP1 hyperactivation, and reduced CREB-dependent pro-survival signaling provides a mechanistic explanation for the Huntington’s disease-related pathways summarized in Figure 2.

Ruijs-Aalfs syndrome represents a distinct but highly informative DNA-repair disorder caused by pathogenic variants in SPRTN, which encodes a DNA-dependent metalloprotease required for proteolytic repair of DNA-protein crosslinks (DPCs) (Weickert et al., 2023; Lopez-Mosqueda et al., 2016). Defective SPRTN function leads to persistence of protein-linked DNA breaks, replication stress, chromosomal instability, premature aging, and cancer predisposition (Weickert et al., 2023; Lopez-Mosqueda et al., 2016). Although Ruijs-Aalfs syndrome is not a common neurodegenerative disease, it illustrates how failure of a specialized genome-maintenance pathway can produce systemic premature-aging phenotypes.

## Effect of exercise in CNS-related disorders

Because the focus of this review is DNA damage, DNA repair, aging, and neurodegenerative disease, psychiatric outcomes such as depression, anxiety, schizophrenia, autism, and bipolar disorder are not discussed in detail. The following section therefore focuses on CNS-related disorders and neurobiological outcomes for which exercise has been linked to oxidative stress, neuroplasticity, neurotrophic signaling, or DNA repair-relevant pathways (Nay et al., 2021).

Physical activity treatment is recognized to have a direct, beneficial influence on degenerative brain conditions and brain damage caused by injury. PD, a degenerative neurological disorder characterized by impairment of movement and cognitive abilities, is linked to damage of cells in the brain (Prakash et al., 2014; Rai et al., 2016; Yadav et al., 2017). Without a doubt, the primary pathological features of PD include the demise of cells in the basal ganglia, which can impact as much as 70% of the dopaminergic neurons in the substantia nigra region of the brain, and the existence of Lewy bodies, which are buildups of the protein *α*-synuclein, in a significant number of the remaining neurons (Balestrino and Schapira, 2020). The earliest indicators that are readily apparent include trembling, stiffness, reduced speed of motion, and trouble with walking. Research conducted in medical settings has confirmed that engaging in physical activity can help to alleviate symptoms in individuals with PD (Schenkman et al., 2018; Frazzitta et al., 2013). AD is a degenerative neurological condition that is both progressive and irreversible. It is characterized by the progressive degeneration and destruction of neurons and synapses in the cerebral cortex and specific regions of the brain, leading to symptoms such as memory loss, anxiety, and confusion (Rai et al., 2020; Singh et al., 2024; Tripathi et al., 2024). It has been scientifically proven that an inactive way of living can result in developing AD earlier. Through their study, Liang et al. discovered that engaging in physical activity can decrease the presence of AD-related indicators (such as Pittsburgh compound-B, tau, and phosphorylated tau) in the cerebrospinal fluid of older individuals without cognitive impairments (Liang et al., 2010). Huntington’s disease is a genetic disorder that leads to the gradual decline of nerve cells in the brain. Unfortunately, there is presently no known cure for this condition, which significantly hinders the functional abilities of those affected and often leads to problems with movement, thinking, and mental health. However, exercise training has emerged as a viable and secure form of treatment for Huntington’s disease patients, with potential benefits to cognition and motor skills (Mueller et al., 2019).

One specific illness that causes damage to the protective sheath surrounding nerves and leads to nerve demyelination and dysfunction is known as Multiple Sclerosis. Common symptoms of this condition include impaired vision, eye pain, fatigue, and coordination difficulties. Recent research has shown that exercise may have the potential to serve as a treatment for individuals with Multiple Sclerosis. Studies have demonstrated that resistance and endurance exercises, as well as aquatic therapy, have beneficial effects on a range of specific symptoms associated with Multiple Sclerosis. As a result, individuals may experience an overall improvement in their quality of life without experiencing any negative consequences (Reynolds et al., 2018). The ability of neurons to change and adapt their synaptic connections is known as synaptic plasticity, and it is influenced by the level of neuronal activity. This results in alterations in the effectiveness and efficiency of the existing synaptic connections between neurons (Magee and Grienberger, 2020). The changes, which can range in length from brief moments to extended spans of several hours or even days, greatly influence essential functions such as learning and memory, and assist in the healing of the brain following injury (Mateos-Aparicio and Rodríguez-Moreno, 2020). Synaptic plasticity is the process by which synapses can either become stronger over time, known as long-term potentiation, or weaker over time, long-term depression (Bettio et al., 2019). Over the course of forty years, there has been a thorough investigation and description of synaptic plasticity. However, the complex biochemical mechanisms behind this process are not completely comprehended, and additional studies are necessary to gain insight into how synaptic plasticity manifests in practical scenarios, for instance, while acquiring knowledge or in situations involving neurodegenerative disorders (Cheyne and Montgomery, 2020). Doing physical activity will boost synaptic plasticity. Multiple studies on animals have demonstrated the ability of consistent exercise to enhance memory and learning abilities, as well as combat cognitive decline related to aging (Lourenco et al., 2019; de Sousa Fernandes et al., 2020). Based on this information, we recently carried out a research project to evaluate how a 12-week aerobic exercise routine, three times a week, affected the synaptic plasticity of 4-month-old mice. This was accomplished by measuring the brain’s electrical activity and examining the structure of the hippocampus in great detail. The results demonstrated that participating in aerobic exercise not only increased synaptic plasticity in comparison to a sedentary group of mice, but it also had positive effects on the ultrastructure of the hippocampus, which aligns with previous studies. The positive effect of aerobic training on learning and memory functions was clearly demonstrated by the well-preserved structure of crucial elements including mitochondria, neurofilaments, and neurotubules (De Domenico et al., 2017; Cariati et al., 2021c).

The impacts of engaging in physical activity on the well-being of the brain are highly prominent in experiments using mice as subjects, particularly in cases where aging is a factor. These studies have revealed that active mice show improvements in cognitive function compared to their sedentary counterparts. A recent study conducted by Tsai et al. involved utilizing a group of mice of varying ages in a treadmill exercise routine, with findings indicating that regular physical activity is a powerful defense against age-related alterations in the brain. These changes include a decline in dendritic length and complexity, as well as a decrease in spine density within CA1 neurons (Tsai et al., 2018). In particular, a 6-week regimen of moderate-intensity running on a treadmill was found to improve the ability for neurons in the hippocampus to change and adapt, and to maintain the complexity of dendrites in all three groups of subjects. Additionally, it was observed that this exercise effectively restored cognitive functions, such as memory and learning, in older mice (Tsai et al., 2018). In a comparative research, Li et al. investigated the effects of six consecutive days of treadmill running on the cognitive capabilities of older mice. The results showed a significant improvement in their spatial memory and a notable decrease in pro-apoptotic signals in the hippocampus (Li et al., 2016). Collectively, these findings indicate that engaging in aerobic exercise is important for stimulating the neural changes that facilitate learning and memory. Furthermore, it also serves to safeguard against the loss of neurons and cognitive impairment that comes with age. Remarkably, there was a remarkable enhancement in the adaptability of synapses after being subjected to well-designed whole-body vibration (WBV) routines, taking into account the vibration frequency, duration of exposure, and rest periods between training sessions.

To gain a deeper understanding, we subjected four-month-old mice to three different WBV procedures. Following a total of 36 sessions, we assessed changes in hippocampal synaptic plasticity using electrophysiological techniques to determine which protocol resulted in the greatest enhancements in cognitive function and memory (Cariati et al., 2021b). The new WBV method, which involved shorter periods of vibration and longer breaks between sessions, was found to have a more significant impact on the hippocampus compared to the control group. Strikingly, even the 24-month-old mice showed improved synaptic plasticity and a reversal of cognitive decline after following the WBV protocol, despite being sedentary (Cariati et al., 2021b). Ultimately, the advantages of WBV training have been witnessed in middle-aged mice, exhibiting enhanced synaptic plasticity and considerable muscle fiber diameter growth, while also effectively maintaining cellular ultrastructure. This indicates that WBV training could be a beneficial approach in delaying symptoms associated with a sedentary way of living (Cariati et al., 2021a). Research has shown that exercise has remarkable positive impacts on human beings, especially in older individuals. Regular physical activity has been linked to enhancements in memory and executive function in the brain, reduced cognitive decline related to aging, and increased safeguarding against atrophy in brain regions that affect higher cognitive functions (Firth et al., 2018). Studies involving the elderly have specifically shown the impact of different forms of physical activity, such as endurance exercises, balance exercises, and practicing new ways of walking on the structure, functioning, and connectivity of the brain (Martínez-Velilla et al., 2019). Specifically, engaging in aerobic exercises has been scientifically shown to increase the volume of both the grey and white matter in the prefrontal cortex among elderly individuals. It can also enhance the size of the hippocampus and medial temporal lobe. This can lead to better spatial memory and a decreased chance of experiencing cognitive decline (Colcombe et al., 2006; Erickson et al., 2009). In a study involving 120 elderly individuals, Erickson and his team conducted a randomized controlled trial that revealed the potential for the prevention of hippocampal volume loss in late adulthood. By implementing a 1-year aerobic exercise program, a 2% increase in hippocampal volume was observed and it effectively countered the decline typically associated with aging (Erickson et al., 2011).

Exercise has various clinical implications, particularly related to stress response. The noradrenergic neurons in the locus coeruleus (LC) play a crucial role in regulating attention, arousal, and vigilance in response to stress. These stress responses from the LC involve norepinephrine signaling and are also affected by galanin, a regulatory peptide that is produced from the cleavage of preprogalanin. Galanin helps to decrease excessive excitability in neurons and may contribute to the adaptation of noradrenergic neurons to stress. Furthermore, exercise has a significant impact on both galanin and preprogalanin. After 5–6 weeks of exercise, there is a decrease in noradrenaline levels during and after foot shock stress, although there is no noticeable change in the expression of TH mRNA or galanin levels in the LC (Morgan et al., 2015; Soares et al., 1999). As more research is conducted, it is becoming increasingly apparent that exercise has significant impacts on sleep and circadian rhythms in both humans and rodents. However, the specific mechanisms behind these effects are not yet fully understood. While the suprachiasmatic nucleus (SCN) is recognized as the main hub for coordinating circadian processes, other brain structures such as the brain stem also play a role (Morgan et al., 2015; van Oosterhout et al., 2012). Maintaining healthy signaling of leptin and insulin in the hypothalamus plays a crucial role in regulating energy balance, as any disruptions in these processes can lead to an increase in food consumption and subsequent weight gain or obesity. Physical activity has a significant impact on central nervous system measures associated with energy balance and food intake. Studies have shown that rats who engaged in exercise for 2 days to 1 week exhibited reduced consumption of high-fat food, attributed to a decrease in meal size and frequency mediated by the corticotropin-releasing factor (CRF/CRH) pathway in the dorsomedullary hypothalamus. Additionally, there is a notable increase in leptin signaling in the ventral tegmental area, indicating that exercise has a regulatory effect on food choices through the activation of CRF and leptin pathways (Scarpace et al., 2010; Kawaguchi et al., 2005). There are various ways that the hypothalamus may be impacted by exercise and can have a less direct effect on maintaining a healthy metabolism and balance of energy. When there is an excess of stored fat, it can lead to heightened stress on the endoplasmic reticulum, a cell organelle, which can impede the effectiveness of insulin in the liver. This is a key molecular process that can contribute to the development of type two diabetes (Ozcan et al., 2004).

**Figure 3:**
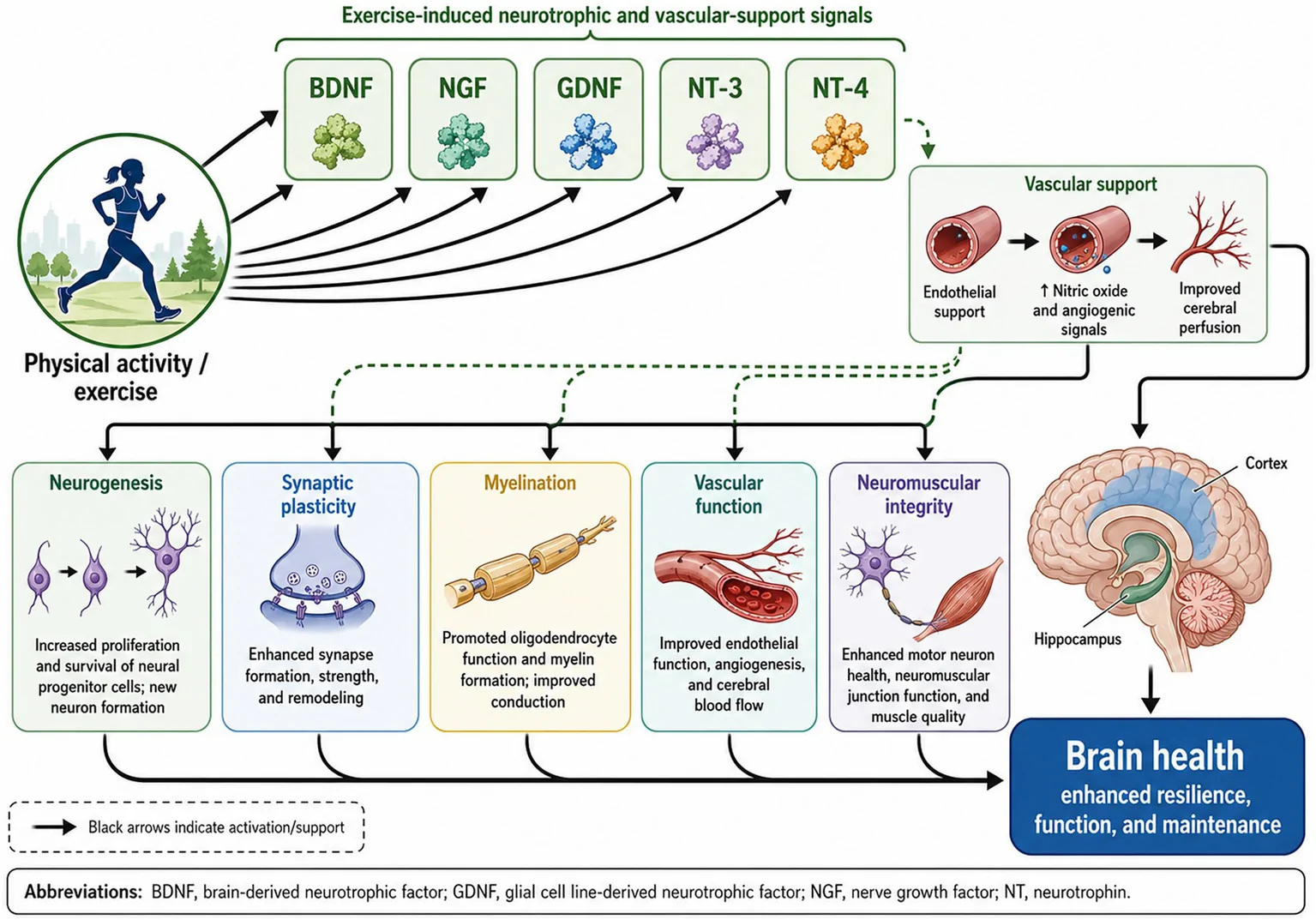
Exercise-induced neurotrophic and vascular mechanisms supporting brain health. Physical activity can increase neurotrophic and vascular-support signals, including BDNF, NGF, GDNF, NT-3, and NT-4, thereby supporting neurogenesis, synaptic plasticity, myelination, vascular function, and neuromuscular integrity. BDNF, brain-derived neurotrophic factor; GDNF, glial cell line-derived neurotrophic factor; NGF, nerve growth factor; NT, neurotrophin.

In conclusion, recent findings indicate that incorporating both physical activity and cognitive assessments into prevention and treatment efforts can significantly enhance their effectiveness in mitigating cognitive deterioration. This further supports the vital role of exercise in promoting better longevity and slower aging processes (Karssemeijer et al., 2017; Law et al., 2020). In short, neurotrophins are crucial for ensuring brain wellness through exercise, as they are key elements in facilitating the brain’s plasticity and ability to adjust. This solidifies their significant role in this aspect (Ribeiro et al., 2021). The presence and intricacy of these factors are important, as they play multiple roles in various biological functions. These include generating new neurons and synapses, forming axons and dendrites, modifying synaptic connections, and preserving the survival of neurons (Vilar and Mira, 2016). Neurotrophins involve in controlling adult neurogenesis, which is the process of generating new neurons in specific areas of the brain during a person’s lifetime. Thus, by producing neurotrophins, physical activity may enhance cognitive function preservation and promote neurogenesis, ultimately preventing age-related cognitive decline (Ma et al., 2017). We looked into various neurotrophins that have been linked to potential neuroprotective effects, with particular emphasis on, nerve growth factor (NGF), neurotrophin-4 (NT-4), brain-derived neurotrophic factor (BDNF), glial cell line-derived neurotrophic factor (GDNF), and neurotrophin-3 (NT-3). This can be seen in the diagram presented in Figure 3.

## Exercise affects DNA damage

**Figure 4:**
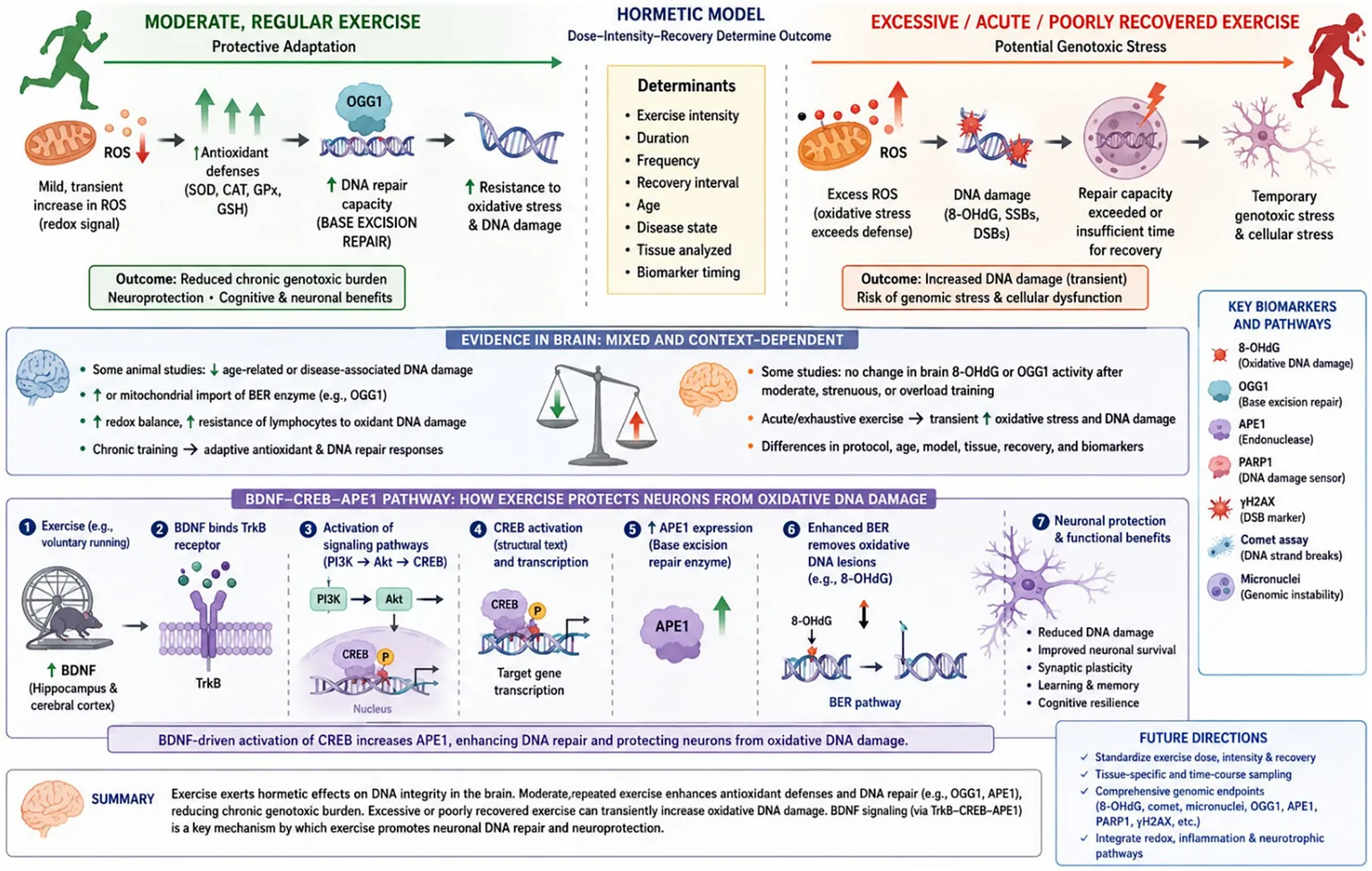
Exercise affects DNA damage through context-dependent hormetic mechanisms in the brain. Regular moderate exercise may induce mild redox signaling that enhances antioxidant defense, base excision repair activity, and resistance to oxidative DNA damage, partly through OGG1- and APE1-related repair pathways. In contrast, acute exhaustive exercise or insufficient recovery may transiently increase reactive oxygen species, DNA lesions, and genotoxic stress when repair capacity is exceeded. Brain-specific findings remain mixed and appear to depend on exercise intensity, duration, recovery interval, age, disease state, tissue analyzed, and biomarker timing. The BDNF–TrkB–PI3K/Akt–CREB pathway may promote APE1 expression and enhance repair of oxidative DNA lesions, supporting neuronal survival, synaptic plasticity, and cognitive resilience. APE1, apurinic/apyrimidinic endonuclease 1; BDNF, brain-derived neurotrophic factor; BER, base excision repair; CREB, cAMP response element-binding protein; DSB, double-strand break; OGG1, 8-oxoguanine DNA glycosylase-1; ROS, reactive oxygen species; SSB, single-strand break; TrkB, tropomyosin receptor kinase B.

The level of DNA damage is closely linked to the ability of brain cells to repair themselves, ultimately involved in reversing brain DNA malfunctions (Figure 4). 8-oxoguanine DNA glycosylase-1 is an essential component of the base excision repair process, specifically responsible for identifying and removing 8-hydroxy-2′-deoxyguanosine from DNA (Iida et al., 2002; de Souza-Pinto et al., 2008). Furthermore, changes in the redox state led to an increase in the gene expression of 8-oxo guanine DNA glycosylase-1 (Adaikalakoteswari et al., 2007).

## Exercise and DNA damage: reconciling protective and genotoxic findings

The relationship between exercise and DNA damage is context-dependent rather than uniformly protective. Regular moderate exercise is generally associated with improved antioxidant defense, increased resistance to oxidative stress, and enhanced DNA repair-related activity in several tissues (Soares et al., 2015; Radak et al., 2009; Koltai et al., 2010; Siu et al., 2011; Radák et al., 2002; Radák et al., 2003; Pittaluga et al., 2015). Mechanistically, these effects may involve increased activity or mitochondrial import of base excision repair enzymes such as OGG1, improved redox balance, and greater resistance of lymphocytes to oxidant-induced DNA damage (Radak et al., 2009; Koltai et al., 2010; Siu et al., 2011; Radák et al., 2002; Radák et al., 2003). These findings support the concept that repeated moderate exercise can reduce chronic genotoxic burden through adaptive redox and repair responses.

However, the brain-specific evidence is less consistent. Some animal studies report that exercise reduces age-related or disease-associated DNA damage, whereas others show no significant change in brain 8-OHdG or OGG1 activity even after moderate, strenuous, or overload training (Vilela et al., 2017; Ogonovszky et al., 2005; Manoharan et al., 2016; Yang et al., 2014; Kim et al., 2010). These apparently conflicting findings may reflect differences in exercise intensity, duration, recovery interval, age, tissue analyzed, disease model, and biomarker timing. For example, acute or exhaustive exercise can transiently increase oxidative stress and DNA damage in muscle or lymphocytes, while chronic training may reduce basal oxidative damage through adaptive antioxidant and repair mechanisms (Ryu et al., 2016).

Therefore, the most appropriate interpretation is a hormetic model. Moderate and repeated exercise may induce a transient redox signal that activates protective adaptation, whereas excessive or poorly recovered exercise may exceed repair capacity and produce temporary genotoxic stress. This distinction helps reconcile studies showing reduced DNA damage after training with studies reporting inconclusive or conflicting brain DNA damage outcomes. Future studies should standardize exercise dose, recovery period, tissue sampling, and genomic endpoints, including 8-OHdG, comet assay parameters, micronuclei, OGG1, APE1, PARP1, and γH2AX.

BDNF is a crucial factor in facilitating the preservation and development of neurons in the brain as it grows. Additionally, it serves a crucial function in facilitating the adjustment of neural pathways in reaction to activity or exposure, resulting in improved learning and memory capacities in grown-ups. The levels of BDNF are decreased in areas influenced by ailments such as Alzheimer’s, Parkinson’s, and Huntington’s. Nevertheless, administering additional BDNF can ameliorate the impaired functioning of brain cells and deterioration observed in experimental models of these conditions. Considering that neurons are vulnerable to oxidative harm in both healthy and neurodegenerative states, researchers investigated the effect of BDNF on neurons’ capability to cope with this specific type of DNA damage (Yang et al., 2014). Upon examination, researchers determined that BDNF has a shielding effect on cerebral cortical neurons from dying due to oxidative damage to their DNA. This method of protection is accomplished by facilitating the restoration of DNA through the promotion of repair. By stimulating the activation of CREB, a protein that binds to particular DNA sequences, BDNF initiates the production of APE1, a vital enzyme that performs an important function in repairing lesions in DNA through the base excision mechanism. The protective effects of BDNF were nullified when either APE1 or TrkB, a receptor for BDNF, was suppressed through RNA interference. The impact of BDNF on promoting the activity of CREB and increasing the expression of APE1 is negated by the interference of TrkB shRNA and inhibitors targeting TrkB, PI3 kinase, and Akt kinase. Through the use of a voluntary running wheel, levels of BDNF are notably augmented, and along with this, there is activation of CREB and heightened expression of APE1 in the hippocampus and cerebral cortex in mice. This indicates that engaging in physical activity could provide a novel means of protecting neurons from oxidative harm to their DNA. The research reveals an unacknowledged finding that BDNF can bolster DNA mending and trigger the production of the DNA-repairing enzyme APE1 (Yang et al., 2014) (Figure 4).

## Impacts of moderate vs. intense physical exercise on DNA integrity

Moderate-intensity physical activity is commonly recommended as a way to decrease the likelihood of acquiring various acute and chronic illnesses. Nonetheless, there is evidence that intense exercise may increase inflammation and cause damage through the production of free radicals, potentially outweighing the undeniable advantages of consistent, moderate physical activity. As ROS are produced during high-intensity workouts and have been shown to affect the stability of DNA, we will explore the existing research on the relationship between intense exercise and DNA damage (Danese et al., 2017b).

Similarly, professional athletes have shown to have a lower incidence of both all-cause and cardiovascular mortality in comparison to the general population. It is worth noting that the American College of Sports Medicine also recognizes the immense advantages of regular exercise for adults, highlighting that the potential dangers during intense physical activity, such as temporary spikes in coronary heart disease and musculoskeletal issues, are outweighed by the overall benefits (Garatachea et al., 2014; Garber et al., 2011). Strenuous exercise, particularly in individuals who are not used to it, has been mainly linked to the formation of blood clots due to a temporary surge in blood clotting caused by hypercoagulability. The key changes in the blood clotting process that contribute to this state are heightened production of the enzyme thrombin, heightened responsiveness of platelets, and heightened function of coagulation factors (Lippi and Maffulli, 2009). It appears that muscle damage is caused by the harmful effects of oxygen radicals. In addition, there is strong evidence that intense physical activity can lead to multiple negative effects, such as elevated levels of lipid peroxidation, glutathione oxidation, protein oxidation, and damage to nucleic acids. These effects can be largely attributed to an imbalance between oxidative stress and the ability of antioxidant systems to combat it (Lee et al., 2015; Sachdev and Davies, 2008). In contrast to intense workouts, engaging in routine, moderate physical movement has a different outcome. It can actually increase the activation of DNA repair genes, likely due to an increase in certain DNA repair enzymes like oxoguanine DNA glycosylase (Danese et al., 2017a).

A study conducted by Ogonovszky et al. aimed to examine whether exercise at moderate (MT), strenuous (ST), or overload (OT) levels can lead to changes in brain DNA damage by measuring the levels of activity of 8-OHdG in the brain of rats. The results showed that oxidative damage to DNA did not significantly differ with exercise, and there was no significant change in the activity of 8-oxoguanine DNA glycosylase (OGG1), a DNA repair enzyme, with exercise training (Ogonovszky et al., 2005).

It is a valid inference that engaging in medium to high levels of physical activity causes a degree of DNA damage that is temporary and directly linked to the intensity of the exercise. This harm can be attributed to the direct impact of free radicals on nucleic acids and is effectively repaired within 24 to 72 h in the body. As long as there is adequate rest time between sessions of intense exercise, there should not be any lasting negative effects on the athlete’s well-being. Consistently increasing exercise load in a gradual manner also appears to be the most advisable method for avoiding instability in DNA (Danese et al., 2017b).

## Mechanism of physical activity beneficial effects

Engaging in both moderate and vigorous physical activity offers numerous health benefits. To combat the harmful effects of oxidative stress on the body, individuals of all ages, but especially those in older age groups, can greatly benefit from consistently incorporating physical exercise into their routines. Regular physical activity over time can help mitigate the detrimental effects caused by free radicals. While reactive species are typically associated with negative biological events, they actually play a crucial role in cellular development and functioning. In fact, cells have evolved mechanisms to utilize these reactive species as biological signals. They serve as subcellular messengers in important molecular signaling processes and regulate the activation of enzymes and genes. ROS also play a role in cellular immune response and drug detoxification, as well as being essential for vasodilation, optimal muscle contraction, and initiation of apoptosis. Studies have shown that ROS are produced during exercise and can influence muscle contraction. Modest levels of ROS intake can actually lead to an increase in muscle force. However, excessive ROS production during intense physical activity can contribute to the development of acute muscle fatigue (Reid, 2001). Regular physical exercise not only boosts antioxidant defenses but also decreases lipid peroxidation levels in both young and older adults. In fact, seniors who engage in physical activity display antioxidant capabilities and levels of lipid peroxidation comparable to those of inactive young individuals, underscoring the significance of consistent physical exercise in slowing down age-related degeneration (Bouzid et al., 2018).

Regular physical activity and maintaining an active lifestyle have been proven to have positive effects not only in preventing oxidative stress, but also in providing primary and secondary protection against different health conditions. These include cardiovascular disorders, type II diabetes, metabolic syndrome, and neurodegenerative diseases like Alzheimer’s. The advantages of exercise can also be seen through the production of myokines, which have various effects on the body, including auto-, para-, and/or endocrine functions. These myokines, which include cytokines, interleukins like IL-6, and other peptides, are released by muscle fibers and play a crucial role in safeguarding against low-grade inflammation-related illnesses such as atherosclerosis (Golbidi et al., 2012). The body’s defense against damaging oxidative substances includes both antioxidant enzymes such as superoxide dismutase, catalase, and glutathione peroxidase, as well as non-enzymatic antioxidants like Coenzyme Q10, glutathione, uric acid, lipoic acid, and bilirubin. When the body is exposed to exercise-induced ROS, these antioxidant enzymes are activated and provide protection against various oxidative stress-related illnesses, including cardiovascular diseases, acquired neurological disorders (such as AD and PD), asthma, diabetes, and mitochondrial myopathies. Coenzyme Q10, also known as ubiquinone, is a fat-soluble compound found in most eukaryotic cells, mainly in mitochondria. It works as a component of the electron transport chain and plays a key role in cellular energy production. Its reduced form, ubiquinol, is a crucial antioxidant in the body. While CoQ10 is produced internally, it is also obtained through diet in limited amounts (Simioni et al., 2018).

Various studies have revealed a remarkable diversity among individuals in their reaction to a normal amount of physical activity, with a considerable portion of adults showing no increase in cardiorespiratory fitness (CRF) that surpasses the day-to-day variability observed with regular exercise as recommended by current guidelines. The issue of individual differences in response is highly significant in exercise medicine, impacted by factors such as age, gender, genetics, and exercise intensity. However, there have been limited attempts to measure and analyze these individual variations, which highlights the necessity for further exploration in this area. This paper argues that the importance of individual responses to standardized exercise has been disregarded, although the process of quantifying such responses is intricate (Ross et al., 2019). In experiments with monozygotic twins and nuclear families, researchers found evidence that there is a genetic component that can cause differences in how individuals react to exercise. This was further supported by studies on rodents bred for specific traits. It is not uncommon for certain individuals to not experience favorable results from exercise training, and this applies to both those who show no response and those with inadequate responses. While it is known that genetic makeup can influence how individuals respond to acute exercise and exercise training, no specific genetic factors have been pinpointed as the sole explanation for the varying responses thus far (Vellers et al., 2018). The evaluation of treatment effectiveness is a crucial concern in various fields of medical practice. To implement a tailored exercise regime, it would be necessary to conduct thorough and meticulous studies, utilizing biomarkers to establish a solid framework for creating precise diagnostic methods for assessing exercise outcomes (Ross et al., 2019).

## Functional foods and fitness as genomic guardians: emerging strategies against DNA damage in aging and brain disorders

Functional foods and physical fitness serve a pivotal function as “genomic guardians” by assisting in the protection of our DNA from damage that accumulates over time and contributes to neurological disorders. Functional foods—abundant in antioxidants, vitamins, and bioactive constituents—fortify the body’s innate defense mechanisms against oxidative stress and inflammation, which are principal contributors to DNA damage. Concurrently, consistent engagement in physical fitness enhances cellular repair processes, augments antioxidant capacity, and fosters optimal brain function. Collectively, these emergent strategies present promising, non-pharmacological approaches to sustain genomic integrity, decelerate aging processes, and mitigate the risk or advancement of neurodegenerative diseases. To reduce fragmentation across intervention types, Table 1 integrates the main functional food components, exercise modalities, biological models, DNA damage biomarkers, and proposed mechanisms of genomic protection. Older adults frequently encounter diminished physical activity levels and insufficient vitamin D, which contributes to various health complications. A study evaluated the impacts of vitamin D supplementation (daily administration of 800 IU or monthly administration of 50,000 IU) either as a standalone intervention or in conjunction with a 10-week strength training regimen on chromosomal stability in a cohort of 100 community-dwelling older adults aged 65 to 85 years. All subjects were administered a daily dosage of calcium (400 mg). Notable enhancements in fitness were observed in both walking distance and chair stand assessments. Nevertheless, markers indicative of chromosomal instability exhibited an increase across all experimental groups, with resistance training—not vitamin D supplementation—identified as the factor associated with heightened chromosomal damage and detrimental alterations in antioxidant markers. The frequency of micronuclei was found to correlate with body mass index and body fat percentage, yet not with vitamin D concentrations (Draxler et al., 2023) (Table 1). Aging and sarcopenia are associated with heightened DNA damage and diminished antioxidant defenses. The investigation determined the impact of a 6-month regimen of progressive resistance training (RT), with or without the inclusion of protein and vitamin supplementation (RTS), or cognitive training (CT) on DNA damage in a cohort of 105 institutionalized elderly individuals (ages 65–98 years). DNA damage exhibited an initial increase following 3 months in the RT and RTS cohorts; however, resilience against oxidative DNA damage exhibited a positive trajectory over time within both the RT and CT cohorts. The activities of antioxidant enzymes (specifically catalase and superoxide dismutase) demonstrated a significant increase solely in the RT and RTS cohorts. No statistically significant differences emerged among the groups overall, indicating that both RT and CT contribute to enhanced resistance against oxidative DNA damage, while supplementation does not confer additional advantages (Franzke et al., 2015a).

**Table 1:** Effects of exercise and nutritional supplementation on chromosomal damage and genomic stability in elderly adults.

Functional food/component	Exercise type	Model/context	Main DNA damage or aging-related outcome	Proposed mechanism	Interpretation	Ref.
Polyphenols/flavonoids, including antioxidant-rich plant foods	Moderate aerobic or resistance exercise	Aging and neurodegenerative disease-related models	Reduced oxidative burden; potential reduction in oxidative DNA lesions	NRF2 activation, antioxidant enzyme induction, reduced ROS-mediated DNA damage	Mechanistically plausible; direct DNA repair endpoints remain limited	Bettio et al. (2019), Simioni et al. (2018)
Omega-3 fatty acids	Home-based strength exercise or combined lifestyle intervention	Older adults; biological aging context	Slower DNA methylation clock progression in DO-HEALTH subgroup	Anti-inflammatory effects, membrane stabilization, epigenetic modulation, possible reduction in inflammaging	Strong recent human evidence for additive biological-aging effects, but not direct BER measurement	Bischoff-Ferrari et al. (2025)
Vitamin D	Resistance training or home-based strength exercise	Community-dwelling older adults	No consistent direct reduction in chromosomal instability in earlier trial; additive epigenetic-aging benefit when combined with omega-3 and exercise	Immunometabolic regulation; possible modulation of oxidative stress and epigenetic aging	Effects appear context-dependent and stronger in combined interventions	Draxler et al. (2023), Bischoff-Ferrari et al. (2025)
Vitamin E	Eccentric or exhaustive exercise	Young and older healthy men	Reduced lipid peroxidation and muscle damage markers; no clear direct effect on 8-OHdG	Antioxidant buffering of exercise-induced lipid peroxidation	May reduce oxidative stress, but DNA damage effects are inconsistent	Sacheck et al. (2003)
Vitamin B12 and folate-containing supplementation	Resistance or cognitive training	Institutionalized older adults	Trend toward reduced micronuclei; B12 changes negatively correlated with micronucleus frequency	Support of one-carbon metabolism, nucleotide synthesis, methylation, and chromosomal stability	Potentially useful when deficiency or low status exists	Franzke et al. (2015b)
Fresh red orange juice/polyphenol-rich beverage	Acute exhaustive exercise	Trained older women	Lower exercise-induced MDA and reduced hypoxanthine/xanthine pathway activation; DNA damage/apoptosis markers unchanged	Antioxidant protection against hypoxia/reoxygenation-associated oxidative stress	Protects redox balance more clearly than DNA repair endpoints	Pittaluga et al. (2013)
Creatine monohydrate	Resistance training	Male athletes and older adults	Reduced urinary 8-OHdG and MDA; improved strength and quality of life in older adults	Energy buffering, mitochondrial support, antioxidant-like effects	Good exercise-adjunct candidate for oxidative DNA damage reduction	Rahimi (2011), Amiri and Sheikholeslami-Vatani (2023)
L-arginine	Concurrent aerobic and resistance training	Elderly men	Reduced 8-OHdG and MDA; increased total antioxidant capacity	Nitric oxide-related vascular effects and antioxidant defense enhancement	Combined training plus supplementation showed stronger effects than either alone	Gilani et al. (2018)
Aerobic exercise alone	Treadmill, running wheel, or structured aerobic training	Aging animals and older adults	Improved hippocampal plasticity, memory, and DNA repair-related signaling	BDNF–TrkB-PI3K/Akt-CREB-mediated APE1 induction and BER enhancement	Strongest mechanistic link between exercise and neuronal DNA repair	van Praag et al. (2005), Erickson et al. (2011)
Resistance training alone	Moderate or progressive resistance training	Older adults	Variable effects on micronuclei and oxidative DNA damage; improved antioxidant enzyme activity	Redox hormesis, SOD/catalase induction, muscle-brain endocrine signaling	Beneficial when appropriately dosed; excessive load may transiently increase damage	Draxler et al. (2023), Franzke et al. (2015a), Franzke et al. (2018)
High-intensity interval or vigorous exercise	HIIT/vigorous interval-type exercise	Metabolic and aging-related contexts	Potential redox adaptation but possible transient oxidative DNA damage if recovery is insufficient	Exercise-induced ROS as hormetic signals; mitochondrial and antioxidant adaptation	Should be individualized; excessive intensity without recovery may increase genotoxic stress	Danese et al. (2017a, b), Reljic (2025)

Another investigation analyzed the impact of age—either exceeding or falling short of life expectancy (LE)—on oxidative stress, antioxidant defense mechanisms, and the stability of DNA following a six-month period of resistance training (RT), resistance training supplemented with RTS, or CT among 117 institutionalized elderly individuals (mean age 83.1 years). Each cohort engaged in training sessions biweekly. Noteworthy enhancements in physical performance were observed in both the RT and RTS groups. Participants whose ages surpassed their LE exhibited a more pronounced advancement in an overarching “antioxidant factor” with RT alone when contrasted with RTS and CT, while no significant variations were detected among those whose ages were below LE. Elastic band resistance training predominantly conferred benefits to individuals exceeding LE by augmenting antioxidant defenses and improving DNA stability. Importantly, the inclusion of antioxidant supplementation may attenuate the optimal cellular adaptations elicited by exercise (Franzke et al., 2018). A study examined age-dependent responses to oxidative stress induced by eccentric exercise in a cohort comprising 16 young and 16 older healthy males, who were administered either 1,000 IU/day of vitamin E or a placebo for a duration of 12 weeks. Participants engaged in downhill running both prior to and following the supplementation period. The exercise regimen resulted in an increase in markers indicative of muscle damage (creatine kinase), lipid peroxidation (F2alpha-isoprostanes and malondialdehyde), and a reduction in antioxidant capacity (ORAC), exhibiting similar patterns across both age demographics. Vitamin E supplementation mitigated peak muscle damage in younger males and diminished resting and post-exercise lipid peroxidation in older males. The marker for DNA damage (8-OHdG) remained unaffected by the exercise intervention. Collectively, vitamin E exhibited a modest influence on oxidative stress responses that varied according to age, with no discernible impact attributable solely to age on these alterations (Sacheck et al., 2003).

A work assessed the impact of fresh red orange juice (ROJ) supplementation on oxidative stress in a cohort of 22 healthy, physically trained older women following a singular episode of exhaustive exercise (EE). Throughout a four-week duration, 15 participants ingested ROJ, while 7 individuals acted as the control group. Blood specimens were obtained prior to and subsequent to EE, at both baseline and post-supplementation. EE momentarily perturbed redox equilibrium in both cohorts; however, only the ROJ group exhibited diminished lipid peroxidation (as indicated by lower MDA levels), a reduction in ascorbic acid depletion, and a decrease in the activation of the hypoxanthine/xanthine metabolic pathway following supplementation. Markers indicative of DNA damage and apoptosis did not display any significant alterations. These findings imply that ROJ may confer protective effects against exercise-induced oxidative stress, particularly through mechanisms associated with hypoxia and reoxygenation processes (Pittaluga et al., 2013). In another study, it was assessed the impact of a six-month regimen of progressive resistance training (RT), with or without the incorporation of RTS, as well as CT, on chromosomal damage among a cohort of 97 institutionalized elderly individuals aged 65 to 98 years. All experimental groups exhibited a non-significant tendency towards a decrease in the frequency of micronuclei (MN), which serves as an indicator of chromosomal instability. The RTS cohort demonstrated significant elevations in plasma vitamin B12 and red blood cell folate concentrations, with alterations in B12 levels exhibiting a negative correlation with the reduction of MN frequency. The results imply that both physical and CT may enhance genomic stability in older adults, and that the addition of vitamins B12 and folic acid could potentially mitigate chromosomal damage (Franzke et al., 2015b).

Creatine (Cr), which is endogenously synthesized by the human body and is prevalent in dietary sources such as meat and fish, is frequently employed to augment muscle hypertrophy, strength, and endurance capabilities. Recent empirical investigations indicate that it also possesses antioxidant characteristics. A study aimed to determine the efficacy of creatine supplementation in mitigating oxidative stress and DNA damage resultant from resistance exercise (RE) in male athletes. Over a duration of 7 days, 27 resistance-trained males were administered either creatine (4 × 5 g/day) or a placebo. Post-exercise evaluations revealed that those who received creatine exhibited enhanced athletic performance and diminished indicators of oxidative injury (plasma malondialdehyde and urinary 8-hydroxy-2′-deoxyguanosine) in comparison to the placebo group. This finding implies that creatine may confer protective effects against oxidative stress and DNA damage induced by exercise (Rahimi, 2011). A research examined the influence of an eight-week regimen of concurrent aerobic and resistance training in conjunction with L-arginine supplementation (1,000 mg daily) on markers of oxidative stress (8-OHdG, MDA) and total antioxidant capacity (TAC) in elderly male subjects (approximately 68 years of age). The participants were stratified into four distinct cohorts: training with supplementation, training with placebo, supplementation alone, and a control group. The findings indicated significant reductions in markers of oxidative stress and a notable enhancement in antioxidant capacity across all groups, with the cohort receiving combined training and supplementation exhibiting the most pronounced improvements. Notably, the impact of exercise alone surpassed that of supplementation alone. The study concludes that the conjunction of L-arginine supplementation with concurrent training serves to effectively diminish oxidative stress and enhance antioxidant defenses in older adults (Gilani et al., 2018).

Another work analyzed the impact of resistance training (RT) in conjunction with creatine monohydrate supplementation (CS) on oxidative stress, antioxidant defenses, muscle strength, and overall quality of life among older adults (mean age approximately 68 years). A total of forty-five non-athletic older males and females were systematically allocated into three distinct groups: RT with creatine, RT with placebo, and a control cohort. Throughout a duration of 10 weeks, participants engaged in training sessions three times per week, with creatine administered at a dosage of 0.1 g/kg of body weight per day. The findings indicated that both training cohorts exhibited a reduction in markers of oxidative stress (MDA and 8-OHdG) alongside an elevation in antioxidant enzyme levels (GPX, TAC). Notable improvements in muscle strength and quality of life were observed, with the creatine group demonstrating superior strength enhancements. This study posits that resistance training is efficacious for the augmentation of antioxidant defenses, muscle strength, and overall well-being in older adults, while creatine supplementation may further amplify strength improvements (Amiri and Sheikholeslami-Vatani, 2023). In conclusion, the integration of functional foods with routine physical exercise constitutes a formidable, organic strategy for the preservation of genomic integrity. Such methodologies contribute to the attenuation of DNA damage, the augmentation of antioxidant defenses, and the promotion of healthy aging, thereby presenting a promising potential for the prevention or postponement of age-associated neurological disorders and the enhancement of overall longevity.

## Combined and potentially additive effects of functional foods and exercise on genome integrity

The combined use of functional foods and exercise should be interpreted cautiously. Strict synergy requires evidence that the combined intervention produces a biological effect greater than the effect of either intervention alone, and ideally greater than the expected additive effect in a factorial design. At present, the available literature more strongly supports independent, additive, or potentially complementary effects rather than definitive synergy. Several studies included in this review evaluated exercise alone or supplementation alone, while fewer directly compared combined interventions against both single-intervention arms (Draxler et al., 2023; Franzke et al., 2015a; Sacheck et al., 2003; Franzke et al., 2015b; Pittaluga et al., 2013; Rahimi, 2011; Amiri and Sheikholeslami-Vatani, 2023; Gilani et al., 2018; Franzke et al., 2018).

Exercise primarily acts as a hormetic stimulus. Transient ROS generation during physical activity can activate adaptive antioxidant defenses, mitochondrial remodeling, neurotrophic signaling, and DNA repair-related pathways. For example, aerobic exercise has been linked to BDNF-mediated CREB activation and APE1 induction, providing a plausible mechanism for enhanced base excision repair in neurons (Yang et al., 2014). Functional food components and supplements may support this adaptive environment by reducing excessive oxidative stress, improving inflammatory tone, supplying micronutrients required for nucleotide synthesis and methylation, or supporting mitochondrial function (Simioni et al., 2018; Franzke et al., 2015b; Rahimi, 2011; Amiri and Sheikholeslami-Vatani, 2023; Gilani et al., 2018).

However, combined interventions do not uniformly produce superior genomic outcomes. In older-adult resistance-training studies, nutritional supplementation did not consistently confer additional protection against DNA damage beyond exercise alone, and some findings suggest that antioxidant supplementation may blunt exercise-induced adaptive responses (Draxler et al., 2023; Franzke et al., 2015a; Franzke et al., 2018). Similarly, vitamin E and red orange juice improved selected oxidative stress or lipid peroxidation markers, but did not consistently reduce direct DNA damage markers such as 8-OHdG or micronuclei (Sacheck et al., 2003; Pittaluga et al., 2013). Therefore, the evidence should not be described as uniformly synergistic.

A more accurate interpretation is that functional foods and exercise may act through complementary biological routes: exercise provides the adaptive redox and neurotrophic stimulus, whereas diet or supplementation may shape the metabolic, inflammatory, and antioxidant context in which this adaptation occurs. Future studies should use factorial designs that compare exercise alone, nutrition alone, combined intervention, and control groups while measuring direct genomic endpoints, including 8-OHdG, micronuclei, OGG1, APE1, PARP1 activity, DNA methylation clocks, BDNF signaling, and cognitive or frailty-related clinical outcomes.

## Limitations

Although there is an increasing body of evidence that supports the advantageous impacts of functional foods and physical exercise on the integrity of DNA and aging-related diseases, various limitations impede the current comprehension and clinical application of these findings. Initially, a significant portion of the mechanistic evidence is derived from *in vitro* and animal research, which may not adequately represent the intricacies of human physiology or the progression of disease, especially in the context of neurodegenerative disorders. Human clinical trials are relatively scarce, frequently of brief duration, and exhibit considerable variability regarding dietary elements, exercise regimens, and biomarkers evaluated, thus complicating the formulation of standardized conclusions. Moreover, the bioavailability and metabolic variability of bioactive constituents in functional foods can markedly affect their effectiveness, and individual responses may differ owing to genetic, epigenetic, and microbiome-related influences. Likewise, the benefits derived from exercise may differ according to factors such as age, sex, initial health status, and adherence to prescribed protocols, thereby complicating the determination of optimal strategies for diverse populations. Third, numerous investigations do not explicitly quantify DNA damage or the associated repair mechanisms, instead depending on indirect markers of oxidative stress, which constrains the capacity to delineate causal relationships. Moreover, there is a deficiency of longitudinal studies that monitor DNA integrity along with cognitive or neurological outcomes over an extended period, which is crucial for comprehending the long-term implications of these interventions on the risk and progression of neurodegenerative diseases. Lastly, the synergistic interactions between dietary habits and physical exercise remain insufficiently examined, with the majority of studies focusing on these variables in isolation. A more comprehensive and interdisciplinary approach to research is imperative for a thorough understanding of how these elements interact to affect genomic health and the trajectories of aging.

## Conclusions and key message

Functional foods and consistent physical activity present themselves as promising, non-pharmacological strategies capable of modulating genomic integrity and augmenting repair mechanisms integral to the aging process and the prevention of neurodegenerative disorders. The bioactive constituents present in select alimentary sources—such as polyphenols, carotenoids, and omega-3 fatty acids—demonstrate antioxidant, anti-inflammatory, and epigenetic properties that play a vital role in maintaining genomic stability. In a similar vein, various modalities of physical exercise, particularly aerobic and resistance training, can enhance intrinsic antioxidant systems, promote DNA repair mechanisms, and mitigate oxidative damage at the cellular level. Collectively, these lifestyle determinants may provide an additive or complementary framework for reducing genotoxic burden associated with aging and neuronal degeneration. Evidence derived from both preclinical and clinical investigations indicates that the incorporation of functional nutrition and physical exercise into quotidian routines may postpone the onset or progression of neurodegenerative diseases by safeguarding the genomic structure and enhancing cerebral health. The principal assertion is unequivocal: the restoration and maintenance of the integrity of our DNA—our biological framework—may not necessitate intricate interventions, but instead, a methodical synchronization of dietary practices and physical activity. Future inquiries should concentrate on mechanistic explorations, individualized interventions, and longitudinal clinical outcomes to optimally exploit the genomic advantages of these readily available lifestyle modifications.
